# Human Milk Banking-Facts and Issues to Resolve

**DOI:** 10.3390/nu2070762

**Published:** 2010-07-13

**Authors:** Willemijn E. Corpeleijn, Marijn J. Vermeulen, Ineke van Vliet, Caroline Kruger, Johannes B. van Goudoever

**Affiliations:** 1 The Erasmus MC-Sophia Children’s Hospital, Dr. Molewaterplein 60, 3015 GJ Rotterdam, the Netherlands; Email: w.corpeleijn@erasmusmc.nl (W.E.C.); m.j.vermeulen@erasmusmc.nl (M.J.V.); i.vanvliet@erasmusmc.nl (I.V.); carolinekruger68@gmail.com (C.K.); 2 Vu Medical Centre, Department of Pediatrics, De Boelelaan 1117, 1081 HV Amsterdam, the Netherlands; 3 Academic Medical Center-Emma Children’s Hospital, Department of Pediatrics, Meibergdreef 9 1105 AZ Amsterdam, the Netherlands

**Keywords:** premature neonate, formula, donor milk, donor screening, vertical transmission, human milk fortifier, outcome

## Abstract

The number of human milk banks is increasing worldwide. Although the beneficial effects of feeding premature infants with their mother’s milk are well documented, less is known about the effects of feeding these infants with pasteurized donor milk. We propose a randomized trial comparing the effects of a 100% human milk-based diet (human milk supplemented with a human milk-derived fortifier) and a diet (partially) based on bovine milk. In theory, human milk has a beneficial effect on various aspects of human physiology, most of which become apparent after infancy. We therefore propose an extensive follow-up program that takes this aspect into consideration. Other issues concerning the practice of human milk banks need to be addressed as well as optimization of the feeding strategies for preterm infants.

## 1. Introduction

Worldwide human milk banks are re-emerging after closing in the mid-1980s due to the identification of human immunodeficiency virus (HIV) in breast milk. The aim of human milk banks is to provide safe donor milk to (premature) infants so that they can benefit from the advantages of human milk when their own mother is unable to provide milk. The benefits of feeding (premature) infants with fresh milk from their own mothers are well documented [1]. However, pasteurized donor milk does not necessarily exert the same positive effects as raw milk from the mother. The milk banking process (pasteurization, repetitive freeze-thawing and storage) partly inactivates the biological substances responsible for the beneficial effects, thereby diminishing the nutritional and protective value of human milk [2]. However, there are additional differences between donor milk and mother’s own milk that have to be taken into consideration. For example, breast milk contains specific secretory immunoglobulins (sIgA) formed through the enteromammary pathway in reaction to pathogens from the environment. While a mother and her child usually share the same environment, this is not the case for the donor mother and the receiving infant. It can therefore be argued that the sIgA found in donor milk are not as well tailored to the infant’s needs compared to those in the milk of the infant’s own mother. This difference might be specifically important for children admitted to the Neonatal Intensive Care Unit (NICU) - an environment with unusual pathogens. Although a graft *versus* host reaction caused by leukocytes in raw donor milk has been proposed, there has been no evidence found supporting the existence of such a phenomenon [3]. Given that (preterm) formula is cheaper than processed donor milk and formula does not pose the (albeit theoretical) risk of vertical transmission of infection, proof of the benefit of human donor milk is needed.

## 2. Evidence of the Benefits of Donor Milk

Two meta-analyses have shown a reduction in the incidence of necrotizing enterocolitis (NEC) in low birth-weight infants that were fed donor milk compared to those fed with formula. However, the infants who were fed donor milk showed slower growth rates in comparison to infants who were fed (preterm) formula. Decreased growth rate is associated with neurodevelopmental impairment [4]. Interpretation of these results is hampered by the fact that pasteurized milk without fortification was given to the infants in seven of the eight studies analyzed [5], which is not a reflection of current practice. Milk from the mother is fortified with additional proteins, minerals and vitamins to meet the infant’s requirements. Furthermore, there was a substantial heterogeneity between the studies, and none of them were blinded studies. Most included trials were performed in the late 1970s and early 1980s, while only one was performed after the year 2000. In a cohort study by Hylander et al. [6], consumption of mother’s milk had a large effect on infectious morbidity. They found a 47% combined infection incidence in the formula group compared to 29% in the human milk group. In addition, in a randomized trial by Narayanan et al. [7], human milk consumption (both milk from the mother and pasteurized donor milk) reduced the incidence of infection. De Silva et al., however, did not find conclusive evidence of the protective effect of human milk on the development of late onset sepsis or other infections in their systematic review [8]. Valuable evidence regarding the effects of the different types of feeding for premature neonates was provided by Lucas and co-workers in two large parallel randomized controlled trials conducted in the United Kingdom in the early 1980s. In the first trial, infants were randomized between receiving donor milk or preterm formula (either as a sole diet or in addition to the mother’s milk). The infants in the second trial were randomized between receiving preterm formula or term formula (either as a sole diet or in addition to the mother’s milk). The results of the two trials were analyzed separately and combined. Infants were followed-up into adulthood, thereby providing information on the long-term effects of human milk feeding. At the corrected age of 18 months, infants were subjected to the Bayley scale of infant development to assess their mental development index (MDI) and psychomotor development index (PDI). Infants who were fed preterm formula (protein: 2.0 g, energy: 80 kcal/100 mL) showed a 6-point advantage in MDI scores and a 14.7-point advantage in PDI scores compared to infants who were fed term formula (protein: 1.45 g, energy: 68 kcal/100 mL) [9]. Interestingly, infants fed donor milk (protein: 1.1 g, energy: 50 kcal/100 mL) had similar outcomes compared to infants fed preterm formula, despite the low nutrient content of donor milk compared to the preterm formula and to the estimated needs of preterm infants [9]. Apparently, human milk contains an entity capable of making up for the low nutrient content. The same infants were also studied at 13-16 years of age to assess the influence of early diet on the development of cardiovascular risk factors. Infants who received banked human milk showed lower C-reactive protein (CRP) levels and LDL/HDL ratios [10] and a lower mean and diastolic blood pressure [11]. These studies suggest that, after premature birth, the type of nutrition in the first weeks of life influences the risk of developing cardiovascular disease in later life. Optimization of early nutrition will therefore have a major impact on future health of infants and seems to be a highly effective preventative measure. 

## 3. Studies to be Undertaken

**Figure 1:**
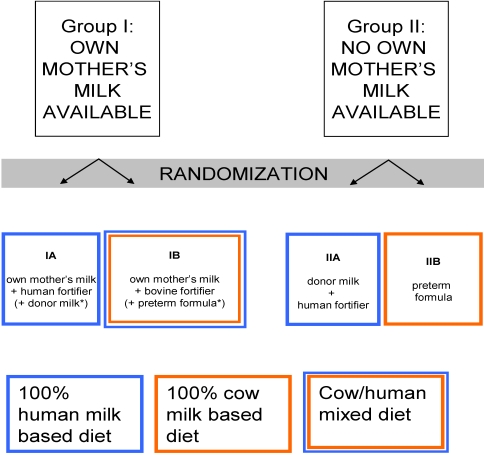
Study design. (Group IA and IB will only be supplemented with donor milk or formula, respectively, if their own mother’s milk supply is insufficient).

Conducting studies on the effects of donor milk in comparison to mother’s milk or formula is complicated for various reasons. First, from an ethical point of view, it is not possible to randomize infants who receive their mother’s milk between formula and donor milk. It is known that mothers from higher sociodemographic groups are more likely to breastfeed their infants [12]. Sociodemographic factors such as household income are known determinants of the development and physical health of children [13] and should therefore be considered confounders in research on the effects of breastfeeding. However, controlling for these confounders might not be sufficient. Secondly, the staff at hospitals where it is common practice to feed premature neonates with donor milk when their own mother’s milk is unavailable considers it to be unethical to randomize infants between donor milk and preterm formula. This kind of research, therefore, needs to be conducted in hospitals in which donor milk is not the standard choice of nutrition for infants not receiving their own mother’s milk. In our and most other hospitals, the majority of mothers delivering a very low birth-weight (VLBW) infant initially express breast milk, but a large proportion of them are unable to provide sufficient amounts of milk to feed their infant during the full admission period, especially not when their infant starts to need larger volumes of milk. These infants therefore receive a mixture of their own mother’s milk and preterm formula or donor breast milk. Basically, two questions need to be answered: First, does donor milk have advantages over preterm formula as a sole diet? Second, does donor milk as a supplement to the mother’s milk hold advantages over supplemental preterm formula? If so, is there a dose-dependent effect of human milk? To answer these questions, we propose the study design depicted in Figure 1. Parameters such as growth rate and the incidence of infections in the different groups should be studied; however, many more aspects of the developing premature neonate are worth studying. Several important beneficial effects of human milk only become apparent after infancy. The follow-up of children included in these studies, therefore, needs to continue into adulthood. The different aspects of human physiology that can be influenced by early nutrition should be taken into account when performing follow-up studies. The possible fields include but are certainly not limited to the following: (1) immunity, allergy and gut health. Human milk is thought to have the ability to induce tolerance to allergens, while at the same time serving as a source of passive immunization and inducing a “healthy” gut flora. This induction would lead to a lower incidence of allergies and infections in later life; (2) neurocognitive development. Essential fatty acids present in human milk and the high taurine content could be responsible for the observed favorable neurodevelopmental outcome in infants fed with human milk. However, the underlying mechanisms need to be elucidated; (3) cardiovascular and metabolic consequences. Human milk seems to improve lipid profiles and lower blood pressure, leading to lower cardiovascular risk in later life; and (4) cost-effectiveness of human milk banking should not only be studied in relationship to the expenses made during admission to the NICU but should also be seen in the light of potential healthcare savings in later life.

## 4. Additional Issues to be Resolved

Some practical issues regarding the processing of human milk and feeding of premature neonates need to be addressed.

### 4.1. Method of Pasteurization

Given that viruses and other pathogens can be transmitted by the consumption of infected milk, most, but not all, human milk banks pasteurize donor milk prior to feeding it to (premature) infants. Holder pasteurization (heating at 62.5 °C for 30 minutes, HP) is the most frequently used method of pasteurization in human milk banks and is known to effectively eliminate viruses such as HIV [14], CMV [15] and most bacteria. However, this procedure has profound effects on the nutrients and immunological factors in human milk. New methods such as flash pasteurization (heating the milk to higher temperatures for a short time) seem promising with respect to eliminating pathogens while preserving valuable biological substances and nutrients [16]. More research needs to be performed to ensure the safety of this method and the optimal temperature and duration of the pasteurization process. Although high temperature-short time (HTST) pasteurizers are widely used in the dairy industry, none are tailored for use in human milk banks.

### 4.2. Safety of the Milk

Most human milk banks use the same selection and screening procedures for donor mothers that local blood banks use for selection and screening of blood donors. This seems to be an appropriate practice because breast milk is produced from blood. Nonetheless, it can be argued that procedures for the selection of milk donors should be adjusted on the basis of the properties of human lactation, the practice of milk banks and the vulnerability of the recipient population. In contrast to blood products, donor milk is pasteurized and is administered enterally. Some diseases transmissible by blood transfusion might not be of importance for the safety of human donor milk consumption, whereas others (known and unknown) might be highly transmissible by human milk. The potential risk of transmission of infectious agents through human (donor) milk is a rather unexplored field. One of the discussed screening criteria for blood donors concerns transmission of variant Creutzfeldt-Jakob (vCJD) disease. In The Netherlands, people who have lived in the UK for more than six months between 1980 and 1996 are excluded as blood donors, and maybe, they should be excluded as milk donors as well. However, in countries with a high incidence of bovine spongiform encephalopathy, no cases of vCJD transmitted through human milk have been reported. In addition, cow’s milk is not included as a source of possible transmission of the disease [17], which makes this screening criteria for milk donation debatable as evidence for transmission of vCJD via human milk is also lacking.

On the other hand, other infectious agents that could be transmitted through human (donor) milk might be relevant. In the last few years there has been an outbreak of *Coxiella burnetii* (Q-fever) in The Netherlands. Q-fever is known to be transmitted by consumption of raw milk from infected cattle but is also thought to potentially be transmitted by blood transfusion. Recently, the Dutch blood banks began screening blood donors from endemic areas for this disease. Vertical transmission of Q-fever *in utero*, during delivery, or lactation, has been suggested [18]. Pasteurization (63 °C, 30 minutes) has been shown to eliminate *C. burnetii* effectively from cow’s milk [19]. Whether pasteurized milk from an infected donor poses a risk to the premature newborn is unknown. Further research in this field is necessary.

### 4.3. Method of Fortification

Unfortunately, both mother’s milk and donor milk lacks sufficient nutrients to meet the high metabolic demands of premature neonates. Human milk is therefore fortified by multinutrient supplements, comprising extra protein, fat, carbohydrate, vitamins and minerals. The nutritional needs of infants are usually met through this supplementation. However, as these fortifiers are derived from cow’s milk, it can be hypothesized that these bovine fortifiers are a contributing factor in the development of NEC in preterm infants, as is seen in formula-fed neonates. Theoretically, the suggested protective effects of human milk might be overruled by the directly disrupting effect of cow milk proteins. A very recent study by Sullivan *et al.* [20] seems to confirm this hypothesis. In this study, a commercially available human milk-based fortifier (Prolact+ H^2 ^MF, Prolacta Bioscience, Monrovia, California) was used in a population with a very high background incidence of NEC. The results showed that infants receiving this fortifier in addition to their own mother’s milk and donor milk had a significantly lower risk to die and/or develop NEC compared to infants fed with a bovine milk-based fortifier and preterm formula in addition to their own mother’s milk. With the development of commercially available machines that perform techniques such as ultrafiltration and freeze-drying, it should become possible for human milk banks to produce a human milk-based fortifier. However, the financial costs of these processes are high, and the clinical benefits of a human milk-based fortifier still remain to be confirmed.

In conclusion, with the quality of human milk banking and of artificial formula constantly improving, it seems mandatory to continuously monitor the effects of our practices. Collaboration of human milk banks on research projects and other aspects (e.g., the production of a human milk-based fortifier) will contribute substantially to our knowledge of the optimal feeding strategy for premature neonates. The present feeding strategies do not meet the requirements when both safety and efficacy are considered. A fortified, exclusively human milk-based diet might solve this issue.
